# Immunotherapy for Hepatocellular Carcinoma: Current Status and Future Prospects

**DOI:** 10.3389/fimmu.2021.765101

**Published:** 2021-10-04

**Authors:** Zhuoyan Liu, Xuan Liu, Jiaxin Liang, Yixin Liu, Xiaorui Hou, Meichuan Zhang, Yongyin Li, Xiaotao Jiang

**Affiliations:** ^1^ Department of Immunology, School of Basic Medical Sciences, Southern Medical University, Guangzhou, China; ^2^ Department of Pediatrics, Nanfang Hospital, Southern Medical University, Guangzhou, China; ^3^ R&D Department, Caleb BioMedical Technology Co. Ltd, Guangzhou, China; ^4^ Department of Infectious Diseases, Nanfang Hospital, Southern Medical University, Guangzhou, China

**Keywords:** immunotherapy, hepatocellular carcinoma, HCC, immune checkpoint inhibitors, adoptive cell therapy, vaccine, CAR-T, TCR-T

## Abstract

Hepatocellular carcinoma (HCC) is the most prevalent primary liver cancer with poor prognosis. Surgery, chemotherapy, and radiofrequency ablation are three conventional therapeutic options that will help only a limited percentage of HCC patients. Cancer immunotherapy has achieved dramatic advances in recent years and provides new opportunities to treat HCC. However, HCC has various etiologies and can evade the immune system through multiple mechanisms. With the rapid development of genetic engineering and synthetic biology, a variety of novel immunotherapies have been employed to treat advanced HCC, including immune checkpoint inhibitors, adoptive cell therapy, engineered cytokines, and therapeutic cancer vaccines. In this review, we summarize the current landscape and research progress of different immunotherapy strategies in the treatment of HCC. The challenges and opportunities of this research field are also discussed.

## Introduction

Hepatocellular carcinoma (HCC) is the fifth most commonly occurring cancer and the third leading cause of cancer death globally (1). In 2020, there were approximately 906,000 new cases and 830,000 deaths of primary liver cancer worldwide, most of which were HCC (comprising 75%-85% of cases) (2). Although surgery is now the most effective treatment for HCC, tumor recurrence is quite common following tumor resection, and the age-standardized five-year relative survival rate for HCC is only 18.1% (3). Due to the difficulty of early diagnosis, the majority of HCC patients are diagnosed as an advanced stage at the initial visit and lose the opportunity for curative treatment such as hepatectomy or radiofrequency ablation, making HCC the second leading cause of cancer-related death in adult males due to the lack of effective therapies (4). The two clinically approved targeted therapy drugs, sorafenib and lenvatinib, could only extend the overall survival by 2 to 3 months (5, 6). Therefore, novel HCC treatment approaches are desperately needed.

**Figure 1:**
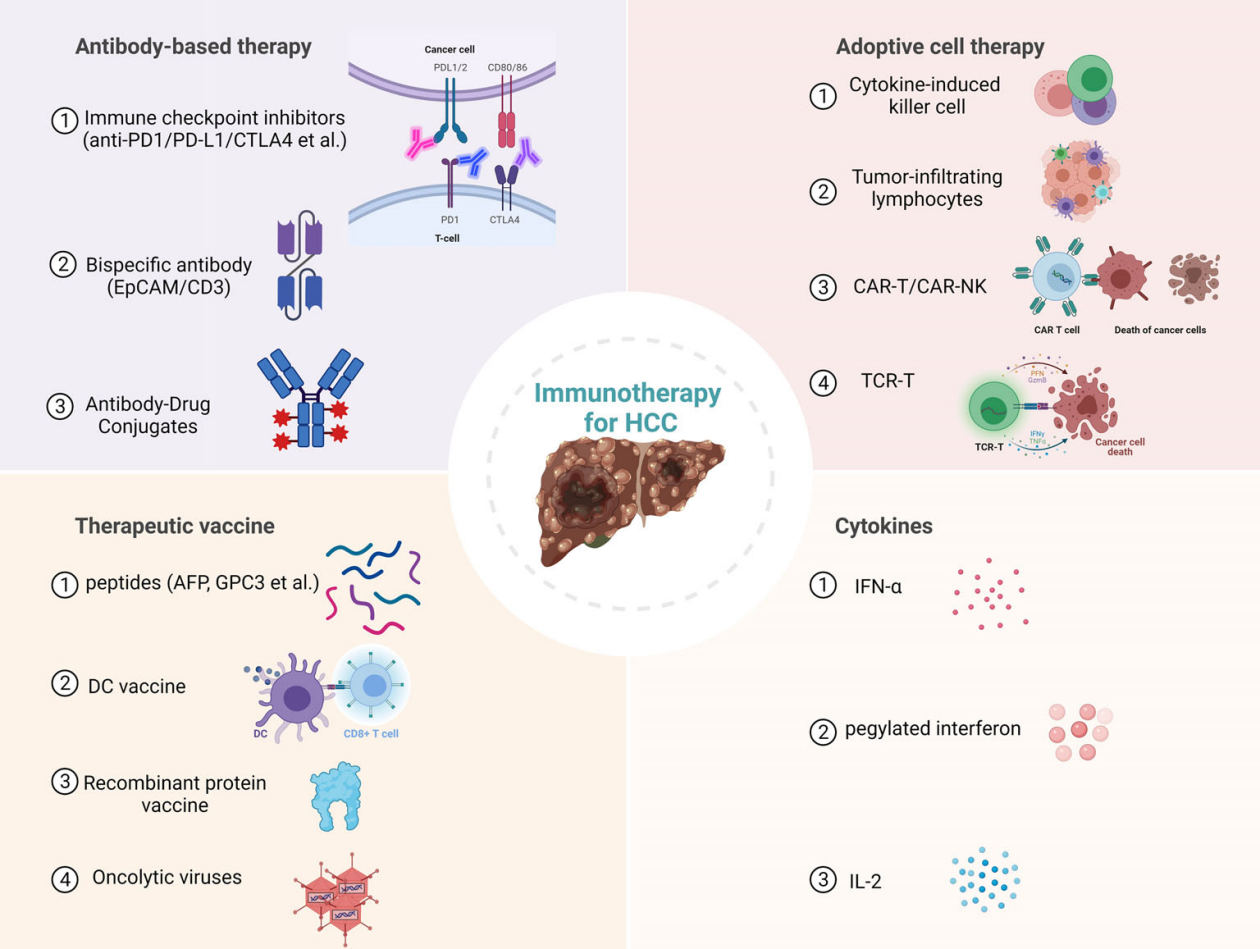
Current immunotherapies for hepatocellular carcinoma (HCC). PD1, Programmed cell death protein 1; PD-L1, Programmed cell death ligand 1; CTLA4, cytotoxic T lymphocyte antigen 4; EpCAM, epithelial cell adhesion molecule; CAR-T, Chimeric antigen receptor T cell; NK, Natural killer cell; TCR-T, T cell receptor engineered T cell; AFP, Alpha-fetoprotein; GPC3, Glypican 3; DC, dendritic cell.

Immunotherapy has been proven effective and safe in treating solid tumors, with long-term survival and tolerable toxicity (7, 8). The liver is an immunologically tolerant organ, uniquely capable of limiting hypersensitivity to antigens from food and bacterial products *via* the portal vein, and capable of accepting liver transplants (9). It is suggested that the development of anti-tumor immunity against HCC is synergistically hindered by this tolerogenic property of the liver and the immunosuppressive tumor microenvironment of HCC. However, the potential of cancer immunotherapy to elicit systemic and durable anti-tumor responses may make it an ideal therapeutic option for HCC, which is characterized by metachronous multicentric occurrence. To date, several immune checkpoint inhibitors (ICIs) targeting cytotoxic T lymphocyte antigen 4 (CTLA-4), programmed cell death protein-1 (PD-1), or its ligand programmed cell death-ligand 1 (PD-L1) have been approved by the U.S. Food and Drug Administration (FDA) for various types of cancers, including HCC (10–12). Other immunotherapeutic strategies, such as adoptive cell therapy, chimeric antigen receptor-modified immune cells, engineered cytokines, and therapeutic cancer vaccines, are matured to clinical trials and bring new hope for HCC patients (13–16). In this review, we first summarize the current landscape of immunotherapy for HCC (**Figure 1**), then discuss this research field’s challenges, opportunities, and future directions.

## Antibody-Based Therapy

### Immune Checkpoint Inhibitors (ICIs)

Immune checkpoints are inhibitory immunoreceptors expressed by effector immune cells that prevent them from becoming overactivated. These inhibitory receptors include but not limited to CTLA-4, PD-1, T cell immunoreceptor with Ig and ITIM domains (TIGIT), T cell immunoglobulin and mucin domain containing-3 (TIM3), lymphocyte-activation gene 3 (LAG3), B and T lymphocyte attenuator (BTLA) (17). HCC and other solid tumors use this physiological mechanism to evade anti-tumor immune responses (18). ICIs are monoclonal antibodies that could block the interaction of immune checkpoint proteins with their ligands, thereby enhance the anti-tumor immune response by preventing the inactivation of T cells and restoring immune recognition and immune attack. At present, the targets of ICIs mainly include PD-1, PD-L1, and CTLA-4 (13). PD-1 is a member of the CD28 family, expressed on the surface of most immune cells, mainly on activated T cells, natural killer (NK) cells, regulatory T cells (Treg), myeloid-derived suppressor cells (MDSC), monocytes, and dendritic cells (DC). PD-1 can bind to its ligands PD-L1 and PD-L2, which are expressed in various tumors, including HCC, to transmit inhibitory signals to T cells and induce the immune escape of tumor cells (19).

In 2017, the PD1 inhibitor nivolumab was granted accelerated approval in the United States for the second-line treatment of patients with advanced HCC after treatment with sorafenib. To date, several exploratory studies of ICIs in treating HCC have been conducted. Pembrolizumab and atezolizumab, targeting PD-1 and PD-L1 respectively, have been gradually incorporated into the treatment guidelines in many countries and recommended as a clinical treatment option for HCC. Nivolumab and pembrolizumab result in a 15-20% rate of objective remissions (including 1-5% complete remissions) that are durable and associated with prolonged survival. In the CheckMate 040 trial, the median duration of response to nivolumab among 48 patients in the dose-escalation cohort was 17 months, and the 2-year survival rate among responders was greater than 80% (20). KEYNOTE-240, a phase III clinical trial testing pembrolizumab following sorafenib treatment in 413 patients compared with placebo, showed statistically prolonged survival (HR 0.78; P=0.023). The progression-free survival and overall survival curves showed that some patients benefited from pembrolizumab in the long term. Nearly 20% of patients who received pembrolizumab remained progression-free for more than one year, compared with less than 7% in the control group (21). The phase III CheckMate 459 trial compared nivolumab with sorafenib in 743 patients naive to systemic agents, patients who received nivolumab lived longer than those who received sorafenib (median survival 16.4 versus 14.7 months, HR 0.85; P=0.07) (22). Longer follow-up of the CheckMate 459 trial confirmed the ability of nivolumab versus sorafenib to increase the rate of long-term survival (29% versus 21% at 33 months) (23). The latest report in European Society for Medical Oncology (ESMO) 2021 Annual Meeting shows that tislelizumab, a humanized monoclonal antibody (mAb) with high affinity for PD-1 demonstrated durable response in patients with previously systemically treated unresectable HCC and was well tolerated. A global, randomized phase 3 trial is ongoing that compares tislelizumab with sorafenib as first-line treatment in adult patients with unresectable HCC (NCT03412773) (24).

CTLA-4 is another member of the CD28 family that is mainly expressed on activated T cells and dendritic cells and is involved in the negative regulation of the immune response after binding to B7 molecules (25). Ipilimumab and tremelimumab are both CTLA-4 inhibitors, of which Ipilimumab is the first immune checkpoint inhibitor approved by the FDA in 2011 for the treatment of patients with advanced skin cancer (26). Ipilimumab is an IgG1 mAb, while tremelimumab is an IgG2 mAb, with different antibody-dependent cell-mediated cytotoxicity (ADCC) and complement-dependent cytotoxicity (CDC) activities (27). A clinical trial in 2013 showed that tremelimumab could effectively play an anti-HCC effect, with a partial response rate of 17.6% and disease control rate of 76.4% (28). With the in-depth investigation of the mechanism of CTLA-4 inhibitors, some scientists believe that the mechanism of CTLA-4 inhibitors is not through the immune checkpoint but by targeted elimination of Tregs in tumors (29). TIM3 is expressed on tumor-infiltrating lymphocytes (TILs) and tumor-associated macrophages (TAMs) of human HCC and negatively regulates the effector function of T cells, whereas its expression on Treg cells results in enhanced suppressor activity (27, 30, 31). The highly expressed TIM3 is associated with less differentiated HCC (32). LAG3 expression is significantly higher on tumor-specific CD4+ and CD8+ TILs than in other immune compartments in patients with HCC. LAG3 has another functional soluble ligand, fibrinogen-like protein 1, which is synthesized by hepatocytes (33). On March 5, 2019, the sialic acid-binding immunoglobulin-like lectin-15 (Siglec-15) was described as a novel immunosuppressive molecule in Nature Medicine by Professor Lieping Chen (34). The latest research shows that Siglec-15 promotes the migration of liver cancer cells by repressing the lysosomal degradation of CD44 (35). The T-cell immunoreceptor with immunoglobulin and ITIM domains (TIGIT) is another immune checkpoint involved in tumor immune surveillance (36). The TIGIT/CD155 pathway inhibits T cell activation by enhancing IL-10 production and diminishing IL-12 by DCs (37). Taken together, these preclinical data support the investigation of TIM3, LAG3, Siglec-15, and TIGIT inhibitors in HCC in combination with PD1 and PDL1 blockade.

Current clinical trial results show that patients treated with ICIs alone have a lower response rate, so the combined use of ICIs and other treatments will be the future direction. In 2020, the results from IMbrave150, a global, randomized phase 3 trial, showed that atezolizumab in combination with the anti-angiogenic drug bevacizumab significantly reduced the risk of death in patients with advanced unresectable HCC and significantly improved the quality of patient survival (38). The combination of pembrolizumab plus lenvatinib, a tyrosine kinase inhibitor (TKI), showed an overall response rate (ORR) of 46%, with complete response (CR) and partial response (PR) observed in 11% and 35% of included patients with unresectable HCC, respectively (39). Similarly, recent preclinical and clinical studies have proved that the combined application of ICIs with transcatheter arterial chemoembolization (TACE), radiofrequency ablation (RFA), and radiotherapy can also promote the efficacy of anti-tumor immunotherapy (40, 41). In addition, camrelizumab combined with the chemotherapy regimen FOLFOX4 is being investigated as first-line therapy for advanced HCC in a phase Ib/II clinical trial (42). A summary of the past three years of clinical trials associated with ICIs therapy for HCC is listed in **Table 1**.

**Table 1:** Clinical trials of ICIs therapy for HCC the last three years (www.clinicaltrails.com).

NCT ID	Phase	Interventions	Country
NCT04943679	1, 2	Anti-PD-1/PD-L1/PEG-IFN-α	China
NCT03638141	2	Durvalumab/Tremelimumab	US
NCT04165174	2	Terepril monoclonal antibody/Apatinib	China
NCT04696055	2	Regorafenib in combination with Pembrolizumab	US
NCT04728321	2	Anti-PD-1/CTLA-4 bispecific antibody AK104/Lenvatinib	China
NCT04444167	1, 2	Anti-PD-1/CTLA-4 bispecific antibody AK104/Lenvatinib	China
NCT04193696	2	Radiation therapy and systemic anti-PD-1 immunotherapy	China
NCT03869034	2	Transarterial Infusion Chemotherapy Combined With PD-1 Inhibitor	China
NCT04974281	1	Anti-PD-1 and Lenvatinib Plus TACE	China
NCT04418401	1	Donafinib Combined With Anti-PD-1 Antibody	China
NCT04814043	2	Anti-PD-1 and lenvatinib plus TACE and chemotherapy	China
NCT04814030	2	Anti-PD-1 Plus Chemoembolization and chemotherapy	China
NCT04273100	2	Anti-PD-1 combined with TACE and lenvatinib	China
NCT03605706	3	Camrelizumab (PD-1 Antibody) in Combination With chemotherapy	China
NCT03839550	2	Apatinib mesylate +PD-1 antibody SHR-1210	China
NCT04564313	1	Anti-PD-1 Antibody Camrelizumab	China
NCT04233840	2	Nivolumab (PD-1 Antibody)	China
NCT04297280	2	Anti-PD-1 Antibody (IBI308) Combined With TACE	China
NCT04229355	3	DEB-TACE plus PD-1 inhibitor	China
NCT03857815	2	Radiation Combined With Anti-PD-1 Antibody (IBI308)	China
NCT04639284	NA	Anti-angiogenic agents plus anti-PD-1/PD-L1 antibodies	China
NCT04518852	2	TACE combined with sorafenib and PD-1 mAb	China
NCT04172571	2	anti-PD-1 antibody AK105 plus anlotinib hydrochloride	China
NCT03939975	2	anti-PD-1 therapy in combination with incomplete thermal ablation	China
NCT04248569	1	DNAJB1-PRKACA Fusion Kinase Peptide Vaccine Combined With Nivolumab and Ipilimumab	US
NCT04802876	2	Spartalizumab (PD-1 inhibitor)	Spain
NCT04191889	2	Hepatic Arterial Infusion combined with Apatinib and Camrelizumab	China
NCT03829501	1, 2	anti-ICOS mAb (KY1044) in combination with anti-PD-L1 mAb (atezolizumab)	US
NCT03652077	1	INCAGN02390 (TIM3 inhibitor)	US
NCT03836352	2	DPX-Survivac, in Combination Cyclophosphamide, Pembrolizumab,	US
NCT03849469	1	XmAb^®^22841 in Combination with Pembrolizumab	US
NCT04709380	3	Radiotherapy Plus Toripalimab	China
NCT04167293	2, 3	Sintilimab and Stereotactic Body Radiotherapy	China
NCT04157985	3	PD-1/PD-L1 Inhibitors	US
NCT04658147	1	Perioperative Nivolumab With or Without Relatlimab	US
NCT03713593	3	Lenvatinib in Combination With Pembrolizumab	US
NCT04629339	2	INCB086550 (Oral PD-L1 Inhibitor)	Bulgaria
NCT04487704	NA	camrelizumab	China
NCT04114136	2	Anti-PD-1 mAb Plus Metabolic Modulator	US
NCT04785287	1, 2	Anti-CTLA4 mAb, Nivolumab, and Stereotactic Body Radiation	US
NCT04116320	1	Focused Ultrasound Ablation and PD-1 Antibody Blockade	US
NCT04740307	2	pembrolizumab/quavonlimab (MK-1308A) plus lenvatinib	US
NCT04665609	3	Thermal Ablation, Anlotinib and TQB2450 (PD-L1 inhibitor)	China
NCT03867084	3	Pembrolizumab (PD-1 inhibitor)	US
NCT04246177	3	lenvatinib and pembrolizumab in combination with TACE	US
NCT03655613	1, 2	PD-1 inhibitor(APL-501 or nivolumab) + c-Met inhibitor (APL-101)	Australia
NCT04052152	2	Anlotinib Hydrochloride Capsules combined with Sintilimab injection	China
NCT04204577	2	Thermal Ablation, Apatinib and PD-1 Antibody SHR-1210	China
NCT04102098	3	Atezolizumab (Anti-PD-L1 Antibody) Plus Bevacizumab	US
NCT04828486	2	Futibatinib and Pembrolizumab	US
NCT03785210	2	Nivolumab (Anti-PD1), Tadalafil and Oral Vancomycin	US
NCT03949231	3	PD1/PDL1 Inhibitor	China
NCT03680508	2	TSR-022 (Anti-TIM-3 Antibody) and TSR-042 (Anti-PD-1 Antibody)	US
NCT03973112	2	HLX10 in Combination With HLX04	China
NCT04912765	2	Neoantigen Dendritic Cell Vaccine and Anti-PD1 (Nivolumab)	China
NCT03859128	2, 3	Toripalimab (PD-1 Antibody)	China
NCT04926532	1, 2	Toripalimab (PD-1 Antibody) Plus Sorafenib	China
NCT03722875	NA	SHR-1210 (PD-1 Antibody) Plus Apatinib	China
NCT04014101	2	Anti-PD-1 Antibody SHR-1210 Combined With Apatinib Mesylate	China
NCT04947826	2	combination therapy of HAIC with PD-1 antibody and VEGF antibody	China
NCT04411706	2	Anti-PD-1 Antibody combined with apatinib and capecitabine	China
NCT03764293	3	Anti-PD-1 Antibody SHR-1210 Combined With Apatinib Mesylate	China
NCT03793725	2	Anti-PD-1 Inhibitor SHR-1210 in Combination With Apatinib	China
NCT04297202	2	Anti-PD-1 Inhibitor SHR-1210 in Combination With Apatinib	China
NCT04393220	2	Combination of PD-1 and VEGFR-2 Blockade	China
NCT04665362	1	Oncolytic Virus M1Combined With Anti-PD-1 Antibody and Apatinib	China
NCT03966209	1	JS001(PD-1 inhibitor)	China
NCT03732547	2	Anti-PD-1 Antibody Combined With PolyIC	China

NA, Not available.

### Bispecific Antibody (BsAb) Therapy

Unlike monoclonal antibodies, BsAbs are prepared mainly by recombinant DNA technology and can specifically bind two antigens or epitopes simultaneously (43). BsAb can directly enhance the activity of immune cells against tumors and can also target immune checkpoints and tumor-associated antigens (TAAs) to reverse immunosuppression in the tumor environment. Therefore, they have more advantages in terms of synergistic effects than monoclonal antibodies and can also mediate a variety of specific biological effects. In most cases, BsAbs recruit and activate immune cells to kill tumor cells by bridging the gap between immune cells and tumor cells (44). Solitomab (AMG110, MT110) is a humanized bispecific EpCAM/CD3 antibody. The anti-EpCAM single-chain variable fragment (scFv) is fused to the anti-CD3 scFv *via* a Gly4Ser linker to form the bispecific T-cell engager (BITE), whose binding to γδ T cells can lead to near-complete lysis of HCC cell lines *in vitro* (45). Another BsAb, Glypican-3 (GPC3)/CD3 BITE, is thought to recruit cytotoxic T lymphocyte (CTL) to eliminate GPC3 + HCC cells (46). In one study, two anti-GPC3 Fab fragments were fused *via* flexible linker peptides to one asymmetric third Fab-sized binding module to form an IgG-shaped TriFab, which could be applied to engage two antigens simultaneously, or for targeted delivery of small and large payloads (47).

## Adoptive Cell Therapy (ACT)

ACT is an immunotherapy that uses the immune cells of the patient or a healthy donor to fight cancer and has recently become an essential tool in the treatment of cancer (48). Compared to antibodies or other targeted drugs, ACT can be activated and replicate ***in vivo*** and has a long-lasting anti-tumor effect. Therefore, ACT is also referred to as a **“**living**”** treatment method (49). ACT is considered a highly individualized cancer therapy because most effector cells are derived from the patient. Because expanded or genetically modified effector cells can recognize and attack tumor antigens, ACT is more specific than chemotherapy (50). ACT clinical trials for the treatment of HCC registered at clinicaltrials.gov in the last three years are listed in **Table 2**.

**Table 2:** Clinical trials of ACT for HCC the last three years (www.clinicaltrails.com).

NCT ID	Target	Phase	Interventions	Country
NCT04121273	GPC3	1	CAR-T	China
NCT02905188	GPC3	1	CAR-T	US
NCT04538313	NA	1, 2	TILs	China
NCT03884751	GPC3	1	CAR-T	China
NCT03899415	HBV antigen	1	TCR-T	China
NCT03980288	GPC3	1	CAR-T	China
NCT03672305	c-Met/PD-L1	1	CAR-T	China
NCT04162158	NA	1, 2	Allogeneic NK cells	China
NCT04368182	AFP	1	TCR-T	China
NCT03971747	AFP	1	TCR-T	China
NCT03993743	CD147	1	CAR-T	China
NCT04011033	NA	2, 3	Autologous iNKT cells	China
NCT03941626	DR5, EGFR vIII	1, 2	CAR-T/TCR-T	China
NCT04951141	GPC3	1	CAR-T	China
NCT04550663	NKG2DL	1	CAR-T	China
NCT03441100	MAGEA1	1	TCR-T	US

ACT, Adoptive cell therapy; GPC3, Glypican 3; CAR-T, Chimeric antigen receptor T cells; TILs, tumor-infiltrating lymphocytes; HBV, hepatitis B virus; TCR-T, T cell receptor engineered T cells; AFP, Alpha-Fetoprotein; iNKT, Invariant natural killer T; DR5, Death receptor 5; EGFR vIII, Epidermal growth factor receptor variant III; NKG2DL, NKG2D ligand; MAGEA1, MAGE family member A1.

### Cytokine-Induced Killer Cell (CIK)

CIK cells are a heterogeneous population of immune cells produced by *in vitro* expansion of human peripheral blood mononuclear cells (PBMC) in the presence of IL-2, IFN-γ, and anti-CD3 monoclonal antibodies (51). CIK cells are mainly composed of natural killer T (NKT) cells, natural killer (NK) cells, and cytotoxic T lymphocytes (CTLs). CIK can recognize tumor cells through the adhesion molecules and lyse tumor cells in a major histocompatibility complex (MHC) independent manner. In a phase I clinical trial, Shi et al. used CIK cells to treat primary HCC and found that the symptoms and characteristics of HCC patients were relieved without significant side effects, indicating autologous CIK cells can efficiently improve the immunological status in HCC patients (52). Clinical trials have also shown that CIK cell therapy can not only be used to treat patients with inoperable primary HCC but also has some effect in treating HCC patients after tumor resection. Takayama et al. reported a clinical trial of CIK treatment in 150 patients with postoperative HCC. They found that the treatment had no significant adverse effects, and the recurrence rate was 18% lower in the treatment group, suggesting that CIK cells therapy could reduce the recurrence rate of patients with postoperative HCC and prolong the recurrence-free survival (53).

Researchers have also made many attempts to combine conventional treatments with CIK cell therapy. TACE combined with CIK cells could prolong progression-free survival in HCC patients compared to TACE alone (54). Wang XP et al. reported that after combined treatment of primary HCC patients with CIK cells and local radiofrequency (RF) hyperthermia, T and NKT cells increased significantly, and alpha-fetoprotein (AFP) decreased from 167.67 ± 22.44 to 99.89 ± 22.05 ng/ml (P = 0.001) (55). Although side effects such as pyrexia, chills, myalgia, and fatigue were associated with CIK therapy in 17% of patients, they were not severe enough to discontinue therapy (56). These data suggest that CIK cells in combination with TACE or RF hyperthermia are safe and effective in treating HCC patients.

### Tumor-Infiltrating Lymphocytes (TIL)

TIL is one of the representative components of the host anti-tumor immune responses, which including regulatory T cells (Treg), NK cells, T cells, and B cells (57). Experiments in mice show that TIL is 50-100 times more effective than lymphokine-activated killer (LAK) cells in treating advanced metastatic tumors (58). The feasibility of TIL therapy was demonstrated in a phase I clinical trial in patients with primary HCC (59). Because TILs are isolated from surgical tumor specimens and can recognize multiple antigens, the tumor-inhibitory effect of TIL is stronger than that of therapies targeting single antigens or mutations. Previous studies have shown that TILs in HCC are rare but may have a significant impact on tumor recurrence and patient prognosis (60). In a randomized clinical trial, adoptive TIL therapy was shown to improve recurrence-free survival after liver resection in 150 patients with HCC (53). Patients with HCC and prominent lymphocyte infiltration who underwent surgical resection had a 38.6% lower recurrence rate and a 34.9% higher five-year survival rate than patients without marked lymphocyte infiltration (61). However, it is difficult to isolate TILs from the tumor tissues of HCC patients and expand them *in vitro*. In addition, only a few patients with HCC can tolerate lymphocyte deletion, which is essential before TIL infusion (62).

### Chimeric Antigen Receptor T Cell (CAR-T)

CAR-T therapy is novel cancer immunotherapy in which T cells are genetically modified to recognize specific TAA and is the current research hotspot of ACT (63). CAR-T cell therapy has achieved encouraging outcomes in the treatment of hematological malignancies. CAR-T cells targeting CD19 and B-cell maturation antigen (BCMA) have been approved by the U.S. FDA for the treatment of acute B-cell lymphocytic leukemia, certain types of lymphomas, and multiple myeloma (64, 65). Due to the heterogeneity of solid tumors, lack of specific targets, and susceptibility to the tumor microenvironment, CAR -T therapy for liver cancer is still in development (66).

**Figure 2:**
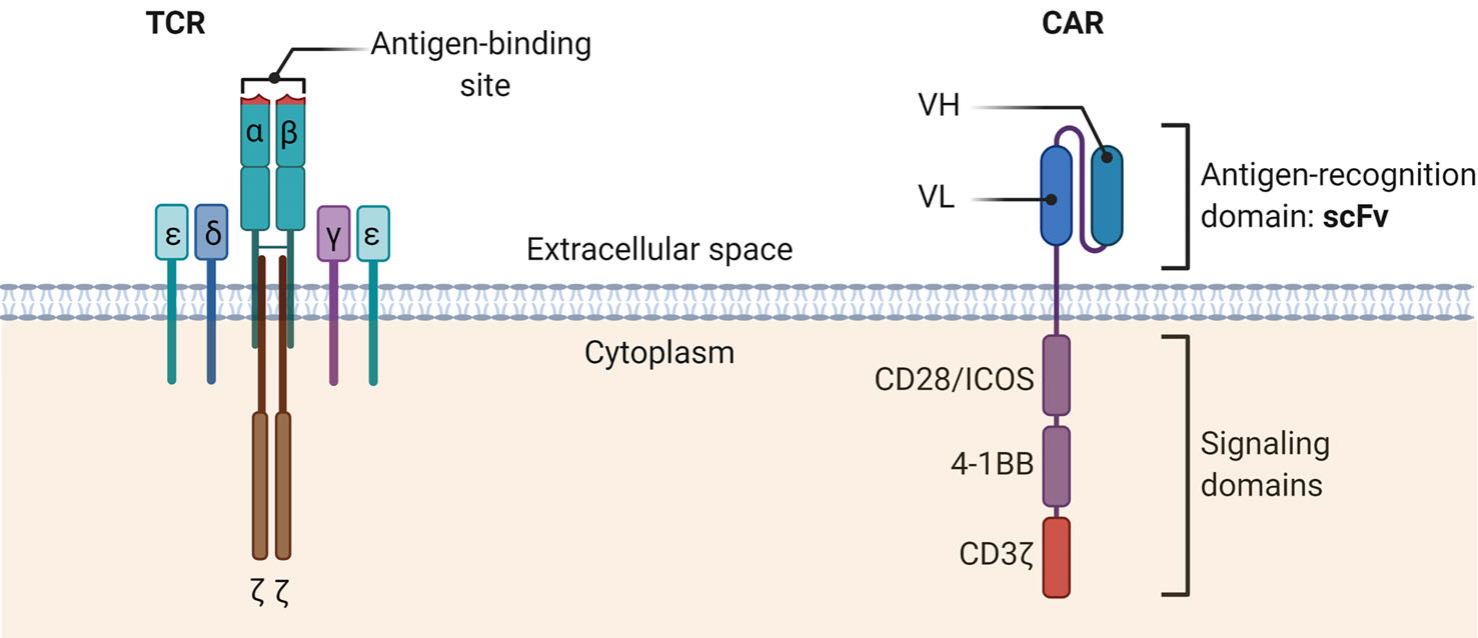
The schematic diagrams of the structures of TCR complex and CAR. The TCR α and β chains bind the MHC-peptide on antigen-presenting cells. Other CD3 molecules, especially the CD3ζ, transmit signals and activate the T cells. TCR, T cell receptor; CAR, Chimeric antigen receptor; scFv, Single-chain variable fragment; VH, heavy chain variable domain; VL, light chain variable domain.

In contrast to the T-cell receptor (TCR) structure of conventional T cells, the CAR structure is independent of the major histocompatibility complex (MHC) antigen presentation, avoids restriction by MHC molecules, and solves the problem of tumor immune escape due to downregulation of MHC (67) (**Figure 2**). To date, a growing number of clinical trials have been conducted to demonstrate the value of CAR-T cell therapy in solid tumors.

GPC3 is a heparan sulfate proteoglycan containing 580 amino acids and is overexpressed in HCC but is not present or shows very low expression in normal tissues (68, 69). Gao et al. constructed for the first time CAR-T cells targeting GPC3 and demonstrated that GPC3 CAR-T cells could effectively eliminate the growth of HCC cells *in vitro* and *in vivo* (70). Recently, our lab reported that by splitting the CAR construct into two parts (split GPC-3 CAR-T cells), HCC tumors could be eliminated with a decreased amount of proinflammatory cytokines (71). Another study established patient-derived xenograft (PDX) HCC models and proved that GPC3 CAR-T cells suppressed tumor growth but with varying efficacy due to different expressions of PDL1 on tumor cells (72). This suggests that the combination of CAR-T therapy and ICIs is a feasible strategy to achieve higher efficacy in eradicating PD-L1-positive HCC.

Alpha-fetoprotein (AFP), a secreted glycoprotein, is highly expressed in the fetus but very low in adults. However, when HCC occurs in adults, AFP is re-expressed (73). Conventional CAR-T cells can only recognize tumor surface but not intracellular antigens. Considering that all intracellular antigens are presented by MHC class I molecules, Liu et al. generated some unique CAR-T cells which can selectively bind to the AFP158-166 peptide-MHC complex, then lyse HLA-A*02:01+/AFP+ tumor cells (74). Meanwhile, they conducted a phase I clinical trial (NCT03349255) successfully evaluating the safety and efficacy of CAR-T cells in AFP-expressing HCC patients. Therefore targeting intracellular antigens with CAR-T cells is a promising strategy for HCC treatment.

c-Met is a tyrosine kinase receptor that can induce hepatocyte proliferation, survival, and regeneration (75). Overexpression of c-Met can promote the development and progression of HCC. Therefore, c-Met is considered a potential target for the treatment of HCC. Jiang et al. generated CAR-T cells targeting c-Met and PD-L1 and found that dual-targeted CAR-T cells exhibited marked cytotoxicity against c-Met+ PD-L1+ HCC cells (76).

Natural-killer group 2 member D ligands (NKG2DL) are expressed in many primary tumors, including HCC, but not in normal tissues (77). Therefore, NKG2DL may provide a useful target for HCC immunotherapy. Recently, Sun et al. constructed novel NKG2D- CAR-T cells that target NKG2DL expressed on HCC cells and found that NKG2D-CART cells specifically lysed HCC cells with high expression of NKG2DL but did not affect the NKG2DL negative cell line (78). The results of the xenograft model also showed that NKG2D-CAR-T cells could successfully inhibit tumor growth *in vivo*.

CD147, a type I transmembrane glycoprotein, was highly expressed in HCC and other solid tumors (79). Zhang et al. introduced Tet-On inducible CD147-CART cells to treat HCC and found that with the supply of Dox, Tet-On inducible CD147-CART cells could lyse multiple HCC cell lines *in vitro* and effectively inhibit the growth of cancer cells in the HCC xenograft model (80). Recently, a phase I study (NCT03993743) was conducted to assess the safety of hepatic artery infusions (HAI) CD147-CART cells for advanced hepatocellular carcinoma.

Other candidates target antigens for HCC CAR-T therapy involve Mucin 1 (81), EpCAM (82), and CD133 (83–85). However, all of the targets mentioned above are TAAs, which are expressed not only in cancer cells but also in normal cells at low levels, therefore causing on-target, off-tumor toxicities in healthy tissues. Finding new specific antigens and improving the efficacy and safety of CAR-T therapy in HCC is the most important task for future researches.

### CAR-NK

In the liver, the proportion of NK cells is significantly higher than in the peripheral blood and spleen. Therefore, NK cell is believed to play an important role in the prevention of HCC and is considered a potential cell therapy resource for the treatment of HCC (86). The strategy used to generate CAR-T cells can also be applied to NK cells to generate CAR-NK cells. In addition, CAR-NK cells can reduce the risk of autoimmune response and tumor transformation because of their shorter lifespan than CAR-T cells (87). Moreover, CAR-NK cells can be produced from a variety of sources, including the NK92 cell line, peripheral blood mononuclear cells (PBMC), umbilical cord blood (UCB), and induced pluripotent stem cells (IPSC). Therefore, CAR-NK cells can be supplied “off-the-shelf”, eliminating the need for personalized and patient-specific products, as is the case with current CAR-T therapies, and reducing the risk of syngeneic xenograft reactions and graft-versus-host disease (GVHD) (88).

In 2018, Yu et al. developed GPC3-specific CAR-NK cells and explored their potential in the treatment of HCC (89). In the study, GPC3-specific CAR-NK cells could induce significant cytotoxicity and cytokine production when co-cultured with GPC3+ HCC cells *in vitro*. Furthermore, soluble GPC3 and TGF-β did not inhibit the cytotoxicity, and no significant difference in anti-tumor activity was observed under hypoxic (1%) conditions. In another study, Tseng et al. utilized CD147 as the target antigen and created CD147-specific CAR-T and CAR-NK cells for the treatment of HCC (90). The results showed that CD147-specific CAR-NK cells could effectively kill various malignant HCC cell lines *in vitro* and HCC tumors in xenograft and PDX mouse models. Importantly, GPC3-synNotch-inducible CD147-specific CAR-NK cells selectively kill GPC3+CD147+, but not GPC3-CD147+ HCC cells and do not cause severe on-target/off-tumor toxicity in a human CD147 transgenic mouse model.

One of the major obstacles to CAR-NK immunotherapy is the lack of efficient gene transfer methods in the primary NK cells. Many recent studies have demonstrated successful transduction of expanded NK cells with retroviral vectors, with efficiencies ranging from 27% to 52% after a single round of transduction (91). However, the insertional mutations associated with retroviral transduction and the deleterious effects on primary NK cell viability are among the most important limitations of this method in a clinical setting.

### TCR-Engineered T Cell (TCR-T)

TCR-T cells are produced by modifying T cells with the gene of exogenous TCRs to specifically recognize the tumor antigen peptides-MHC complex (92). Since all tumor-derived proteins can be processed by proteasomes and presented by MHC, both the tumor surface and intracellular antigens can be targeted by TCR-T cells. Hence, TCR-T therapy should have broader applications than CAR-T.

Hepatitis C virus (HCV) infects approximately 130-150 million people globally and can lead to associated liver diseases, including HCC (93). Spear et al. generated HCV-specific TCR-T cells by genetically engineering T cells with a high affinity, HLA-A2-restricted, HCV NS3:1406-1415-reactive TCR (94). The results showed that HCV-specific TCR-T cells could induce regression of established HCV+ HCC *in vivo*, suggesting HCV-specific TCR-T therapy may be a plausible option for treating HCV-associated HCC.

A smaller percentage of Hepatitis B virus (HBV)-infection-derived HCC tissues retain the HBV gene expression, which can become TCR-T targets. In 2011, Gehring et al. generated HBV surface antigen-specific TCR-T cells from PBMC of chronic HBV and HBV-related HCC patients (95). These HBV-specific TCR-T cells were multifunctional and capable of recognizing HBV-related HCC tumor cells. In addition, a phase I clinical trial was conducted to evaluate the safety and efficacy of HBV-specific TCR-T in preventing the recurrence of HCC after liver transplantation (96) (NCT02686372).

As mentioned earlier, AFP is another HCC-associated TAA. Recently, Docta et al. reported the identification of a human HLAA2/AFP158-specific TCR (97), and a clinical trial using autologous T cells from HCC patients engineered with this AFP-specific TCR has been initiated and is ongoing (NCT03132792). In 2018, we identified multiple HLA-A2/AFP158-specific TCRs from HLA-A2 transgenic mice using an immunization strategy with recombinant lentiviral priming and peptide boosting (98). Human T cells equipped with these TCRs showed potent anti-tumor activity *in vitro* and *in vivo*. Furthermore, systematic X-scan data showed that these TCR T cells have minimal or no cross-reactivity against human cells. A clinical trial using these TCRTs to treat HCC patients has been initiated (NCT03971747).

Other candidates target antigens for HCC TCR-T therapy involve GPC3 (99), New York esophageal squamous cell carcinoma 1 (NY-ESO-1) (100), and human telomerase reverse transcriptase (hTERT) (101). However, due to TCR’s promiscuity, TCR-T cells may cross-react normal tissue MHC-peptide complex, leading to off-target toxicity. Both mouse and human-derived TCRs can produce off-target toxicity. The melanoma-associated antigen (MAGE)-A3/HLA-A1 TCR, although derived from humans, caused significant cardiac toxicity by targeting the cardiac muscle protein titin (102). On the other hand, although NY-ESO-1 TCRT has shown clinical anti-tumor efficacy, most other TCRTs have not been proven effective for patients. Several factors can be considered to improve the anti-tumor effect of TCR-T therapy, including prolonging the survival period of TCR-T *in vivo*, improving tumor infiltration, and preventing T cell exhaustion.

## Therapeutic Vaccine

The therapeutic vaccine is an immunotherapy that introduces tumor antigens into patients in various forms, overcomes the immunosuppressive tumor microenvironment, and then activates the patient’s immune system to fight cancer (103). In 2010, Sipuleucel-T (Provenge) became the first therapeutic autologous vaccine approved by the U.S. FDA for the treatment of men with asymptomatic or minimally symptomatic castrate-resistant metastatic prostate cancer (104). At present, therapeutic vaccines used for HCC mainly include peptides, DCs, and oncolytic viruses. A summary of the past three years of clinical trials concerning therapeutic vaccine therapy for HCC is listed in **Table 3**.

**Table 3:** Clinical trials of therapeutic vaccines for HCC the last three years.

NCT ID	Target	Phase	Interventions	Country
NCT04251117	Neoantigen	1, 2	personalized neoantigen DNA vaccine (GNOS-PV02) and plasmid encoded IL-12 (INO-9012) in combination with pembrolizumab (MK-3475)	US, New Zealand
NCT04912765	Neoantigen	2	Dendritic Cell Vaccine and Nivolumab	Singapore
NCT04248569	DNAJB1-PRKACAfusion kinase	1	Peptide Vaccine Combined With Nivolumab and Ipilimumab	US
NCT03674073	Neoantigen	1	Dendritic Cell Vaccine Combined With Microwave Ablation	China
NCT04147078	Neoantigen	1	Dendritic Cell Vaccine	China
NCT04317248	NA	2	Multiple Signals loaded Dendritic Cells Vaccine	China
NCT04246671	HER-2	1, 2	TAEK-VAC-HerBy vaccine: Modified Vaccinia Ankara-BN (MVA-BN) virus	US
NCT03942328	Streptococcuspneumoniae	1	Autologous Dendritic Cells and Pneumococcal 13-valent Conjugate Vaccine	US

NA, Not available.

In a phase I study, administration of AFP-derived peptides to 15 patients with HCC caused no adverse events and resulted in the generation of T cells with receptors that responded to the peptides. Among the 15 patients, one had a complete response, and eight had a slowing tumor growth. The T cells of the patient who had a complete response expressed a highly functional TCR induced by the peptide vaccines (105). In another phase I clinical trial, a GPC3-derived peptide vaccine was used in 33 patients with advanced HCC and reported that the vaccine was well-tolerated and elicited a high rate of GPC3-specific CTL responses (106). Another phase II study showed that GPC3-positive HCC patients treated with GPC3-derived peptide vaccine as an adjuvant therapy had a significantly lower recurrence rate after one year than patients who received surgery alone (24% vs. 48%, p = 0.047) (107). Multidrug resistance-associated protein 3 (MRP3) is a carrier-type transporter, and its high expression is associated with various cancer cells, including HCC (108). A phase I clinical trial evaluated the safety and immunogenicity of an MRP3-derived peptide as a vaccine in 12 HCC patients (109). The vaccination was well-tolerated, inducing MRP3-specific immunity in 72.7% of patients, with the median overall survival (OS) being 14.0 months (95% CI: 9.6–18.5). When the hTERT-derived peptide was used as a therapeutic vaccine in 14 HCC patients, the induction of hTERT-specific T cells correlated with the absence of HCC recurrence, suggesting a possible role of cellular immunity to hTERT in preventing recurrence (110).

DCs are responsible for T-cell stimulation and anti-tumor immune response enhancement (111). A phase I trial of autologous dendritic cell-based immunotherapy was conducted in inoperable primary HCC patients to evaluate the safety and feasibility. Eight HCC patients were enrolled in this trial, and in one patient, the tumor shrank and showed necrotic changes on computed tomography, whereas in two other patients, serum levels of tumor markers decreased after vaccination (112). Another phase II clinical trial results showed that the DCs vaccine pulsed ex vivo with HepG2 cell lysate was safe and well-tolerated with evidence of anti-tumor efficacy (113). Furthermore, infusion of DC in combination with TACE enhances tumor-specific immune responses more effectively than TACE alone, although the effect is insufficient to prevent the recurrence of HCC (114). Further clinical trials are ongoing, but the results have not yet been announced.

Oncolytic viruses are viral particles engineered to lyse tumor cells and induce anti-tumor immune responses. JX-594 (Pexa-Vec) is currently the main oncolytic virus used in HCC clinical trials (115). JX-594 is a vaccinia virus with disruption of the viral thymidine kinase (TK) gene for cancer selectivity and insertion of human granulocyte-macrophage colony-stimulating factor (hGM-CSF) for immune stimulation (116). Heo et al. reported a randomized phase II clinical trial (NCT00554372) evaluating the feasibility of JX-594 in 30 HCC patients and found that high-dose JX-594 infusion achieved longer median OS compared to the low-dose arm (117). However, in patients previously treated with sorafenib (NCT01387555), the median OS was not significantly different in patients treated with JX-594 (118). Currently, two phase III clinical trials associated with JX-594 in treating advanced HCC is ongoing (NCT02562755, NCT03071094). In summary, although the therapeutic vaccine for HCC shows good prospects, its clinical application still requires further clinical trials to verify its efficacy and safety.

Although therapeutic vaccines have a promising future in treating HCC, some challenges still need to be overcome. First of all, the immunosuppressive tumor microenvironment (TME) of HCC can induce antigen-specific T cell tolerance, resulting in poor vaccine effectiveness. There is a growing need for new therapeutic strategies for HCC vaccines to enhance anti-tumor immune responses by counteracting the immunosuppressive TME. Chemotherapy can enhance the anti-tumor effect of cancer vaccines by overcoming the immunosuppressive TME, improving the cross-presentation of tumor antigens, and increasing the number of effector cells in the TME (119, 120). The combination of appropriately dosed systemic/local chemotherapy with cancer vaccines could be a potentially attractive option for HCC patients. Alternatively, the combination of ICIs and cancer vaccines could be an additional attractive option for HCC patients. Two clinical trials are currently underway using ICIs combined with a kinase peptide vaccine (NCT04248569) or a neoantigen DC vaccine to treat patients with HCC. Another major challenge is that most of the HCC vaccines presented in the current study are based on TAA. TAA is expressed not only on cancer cells but also on normal cells, resulting in an inadequate T-cell immune response and failing to elicit a robust clinical response. Neoantigens are newly expressed antigens in tumors that can be generated from viral proteins, normal cellular proteins, or mutated host genes (121). Since T cells that respond to neoantigens are not negatively selected during thymic maturation and can be primed into potent tumor-killing effector T cells, neoantigens are ideal targets for immunotherapy (122). Given the growing interest in neoantigen-based therapies, many clinical trials of therapeutic vaccines, including three clinical trials for HCC neoantigens, are registered at ClinicalTrials.gov.

## Cytokines

Cytokines are key components of the immune system and play a critical role in the immune response to cancer. Because the immune system is capable of recognizing and destroying cancer cells, there has been great interest in the use of cytokines for cancer treatment in recent decades (123). Interferon-alpha (IFN-α) was the first cytokine approved by the U.S. FDA for the treatment of hairy cell leukemia (HCL) in 1986 (124). High-dose IL -2 was approved in 1992 for the treatment of metastatic renal cell carcinoma (mRCC) and in 1998 for metastatic melanoma (MM). Since initial approval, IFN-α has been extended to follicular lymphoma, melanoma, mRCC in combination with bevacizumab, and acquired immunodeficiency syndrome (AIDS)-related Kaposi’s sarcoma.

A meta-analysis found that IFN-α could decrease mortality and early recurrence rates of HCC following curative treatment but exerted no effect on the late recurrence rate (125). Interestingly, the effect of adjuvant IFN-α on postoperative recurrence differs between HBV-HCC and HCV-HCC cases, indicating different strategies with adjuvant IFN-α should be used to treat HCC with different backgrounds. In another meta-analysis, the effects of adjuvant pegylated interferon (Peg-IFN) therapy on the survival of patients with hepatitis-related HCC after curative treatment were investigated (126). The results showed that adjuvant Peg-IFN therapy could improve recurrence-free survival (RFS) and overall survival (OS) in patients after curative treatment for hepatitis-related HCC without causing severe side effects.

Although IFN-α is gradually being displaced as the first-line anti-tumor drug, the new long-acting Peg-IFN continues to play an important role as a companion drug in HCC treatment (127). A preclinical study using the PDX HCC model has shown that interferon-β (IFN-β), in addition to its antiviral effect, can also exert anti-tumor activity through the JAK-STAT and p53 signaling pathways (128). In addition, IL-2 also has a pleiotropic effect on the immune system, which can increase the proliferation of T cells and activate their anti-tumor action. In patients with inoperable HCC, after treatment with IL-2, the survival rate of patients has increased (129).

However, cytokines as monotherapy has not fulfilled their original promise because parenteral administration of cytokines does not achieve sufficient concentration in the tumor, is usually associated with severe toxicity and induces humoral or cellular checkpoints. To circumvent these obstacles, cytokines are being investigated clinically with newly developed cytokine mutants (superkines), chimeric antibody-cytokine fusion proteins (immunokines), anti-cancer vaccines, and cancer-targeted monoclonal antibodies to enhance their ADCC or to preserve cellular response and anti-cancer efficacy.

## Challenges and Opportunities

The liver is an immunomodulatory organ containing a high density of innate and adaptive immune cells (130). Under physiological conditions, the liver is constantly exposed to intestinal antigens derived from food and microbial products. Accordingly, the liver has intrinsic immune tolerance that allows suppression of inappropriate inflammatory responses (131). The tumor immune microenvironment (TIME) is complex and consists of distinct populations of immune cells that influence response to immunotherapy and patient survival. The TIME of HCC is mainly composed of TAMs, MDSCs, cancer-associated fibroblasts (CAFs), tumor-associated neutrophils (TANs), TILs, DCs, and extracellular matrix (ECM) (132). Compared with other solid tumors, HCC TIME exhibits a more potent immunosuppressive effect, and almost all cell subsets and numerous regulatory mechanisms contribute to HCC progression, posing a major challenge for effective cancer immunotherapy.

In recent years, cancer immunotherapy has made major breakthroughs, and its use in HCC has attracted increasing attention. However, there are still many problems, such as uncertain efficacy, low objective remission rate (OR), numerous side effects, and resistance to the drug even when patients benefit from it. Therefore, improving the tumor immunological microenvironment and balancing the body’s immune response to benefit more patients is an urgent problem and a future development direction for HCC immunotherapy.

Reportedly, the OR of PD-1/PD-1 ICIs alone rarely exceeds 40%, and the OR of nivolumab and pembrolizumab in HCC did not exceed 20% (133). On the other hand, immune-related adverse events (IRAE) is an important reason affecting the widespread use of cancer immunotherapy (134). ICIs can cause inflammatory side effects, including hypophysitis, thyroid dysfunction, and diabetes. CAR-T therapy can cause some severe side effects such as cytokine release syndrome (CRS), neurotoxicity, and even death. In addition, 7%-9% of patients cannot be treated with CAR-T due to failure of CAR-T cell production (135). Other challenges in immunotherapy for HCC and other solid tumors include selecting more specific targets for immunotherapy, how to ensure that ACT cells reach the tumor site more effectively, and how to overcome immunosuppression by the tumor microenvironment.

Another major challenge in immunotherapy for HCC is the lack of markers to predict the effect of treatment. The latest report in the ESMO 2021 Annual Meeting shows that the survival of patients with advanced HCC treated with nivolumab was related to the Child-Pugh (C-P) liver function score at baseline (136). However, other methods of immunotherapy are mostly still in the early clinical stage, and there are no good indicators for predicting the therapeutic effect.

HCC is in a complex immunological microenvironment, so a single immunotherapy method or even immunotherapies alone have a lower remission and survival rate, and multitarget combination therapy should be the focus of future development. In a mouse model, four components of the host immunity consisting of a tumor antigen targeting antibody, an ICI, a powerful T cell vaccine, and a T cell-stimulating cytokine were required to eradicate large established tumors (137). Recently, the data of the phase III clinical trial IMbrave150 showed that atezolizumab in combination with the anti-angiogenic drug bevacizumab significantly reduced the risk of death in patients with advanced unresectable HCC and significantly improved patients’ quality of life, making it the first first-line combination therapy for patients with unresectable advanced HCC (38). The combined use of ICIs against different targets may produce synergistic effects. Similarly, the combined application of immunotherapy with local therapy, such as radiofrequency ablation, radiotherapy, embolization, can also promote the efficacy of cancer immunotherapy (40, 41).

## Conclusion

Although current immunotherapy for HCC has achieved some success, it still faces challenges such as low objective remission rate and adverse treatment reactions. Therefore, comprehensive analysis from multiple aspects to formulate personalized precision immunotherapy schemes for HCC patients, effectively evaluating and predicting the efficacy of immunotherapy, and adopting combined treatment strategies are urgent questions to be answered, and also the future trend of HCC immunotherapy research.

## Author Contributions

All authors listed have made a substantial, direct, and intellectual contribution to the work and approved it for publication.

## Funding

This work was supported by the Natural Science Foundation of Guangdong Province, China (grant number 2020A1515010981, 2020A1515110366), the Science and Technology Program of Guangzhou, China (grant number 202102080193), the President Foundation of Nanfang Hospital, Southern Medical University (grant number 2018C013), and the Outstanding Youth Development Scheme of Nanfang Hospital, Southern Medical University (grant number 2020J003).

## Conflict of Interest

Author MZ works for Caleb BioMedical Technology Co. Ltd, Guangzhou, China.

The remaining authors declare that the research was conducted in the absence of any commercial or financial relationships that could be construed as a potential conflict of interest.

## Publisher’s Note

All claims expressed in this article are solely those of the authors and do not necessarily represent those of their affiliated organizations, or those of the publisher, the editors and the reviewers. Any product that may be evaluated in this article, or claim that may be made by its manufacturer, is not guaranteed or endorsed by the publisher.
